# Qing-Kai-Ling Injection Acts Better Than Shen-Fu Injection in Enhancing the Antitumor Effect of Gefitinib in Resistant Non-Small Cell Lung Cancer Models

**DOI:** 10.1155/2021/9911935

**Published:** 2021-10-04

**Authors:** Ya-Ya Yu, Yan-Juan Zhu, Ying Zou, Zhen-Zhen Xiao, Shuai Shi, Yi-Hong Liu, Xue-Song Chang, Ya-Dong Chen, Hai-Bo Zhang

**Affiliations:** ^1^Department of Oncology, The Second Affiliated Hospital of Guangzhou University of Chinese Medicine, Guangzhou, China; ^2^Department of Oncology, Guangdong Provincial Hospital of Chinese Medicine, Guangzhou, China; ^3^Guangdong-Hong Kong-Macau Joint Lab on Chinese Medicine and Immune Disease Research, Guangzhou, China; ^4^Guangdong Provincial Key Laboratory of Clinical Research on Traditional Chinese Medicine Syndrome, Guangzhou, China; ^5^Department of Integrated Traditional and Western Medicine, Affiliated Cancer Hospital and Institute of Guangzhou Medical University, Guangzhou, Guangdong, China; ^6^Department of Respiration Medicine, Luoyang First People's Hospital, Luoyang, Henan, China; ^7^State Key Laboratory of Dampness Syndrome of Chinese Medicine, Guangzhou, China

## Abstract

Patients with EGFR gene mutation often obtain de novo resistance to epidermal growth factor receptor tyrosine kinase inhibitors (EGFR-TKIs) or develop secondary resistance to EGFR-TKIs after taking EGFR-TKI therapy. Traditional Chinese medicine (TCM) with different treatment principles, in combination with EGFR-TKIs, plays an important role in the treatment of cancers including resistant non-small cell lung cancer (NSCLC). However, inappropriate use of TCM herbs may induce resistance to gefitinib. Therefore, it is of a great value to evaluate which TCM treatment principle should be combined with EGFR-TKIs, and which one should be avoided, and find out the potential mechanisms. The lentiviral transfection assay was used for overexpression of *PIK3CA* mutation gene in PC-9 cells to construct PC-9-PIK3CA-mutation (PC-9-PIK3CA-M) cells. Cell proliferation, apoptosis, and the expression of EGFR/PI3K/AKT and EGFR/RAS/RAF/ERK in PC-9-PIK3CA-M and H1975 cells treated by the typical *cooling-heat* drug, Qing-kai-ling (QKL) and Tan-re-qing (TRQ), or the typical *warming-yang* drug, Shen-fu (SF) and gefitinib treatment, were detected by MTT, Annexin V/PI double labeling, and Western blot assays, respectively. Tumor xenograft and immunohistochemistry experiments were carried out to confirm the *in vitro* findings. PC-9-PIK3CA-M cells were less sensitive to gefitinib, when compared with PC-9 cells. QKL injection and TRQ injection, not SF injection, combined with gefitinib induced significantly increased cell growth inhibition and apoptosis in PC-9-PIK3CA-M and H1975 cells. SF injection antagonized the effect of gefitinib in promoting cancer cell apoptosis. QKL injection and TRQ injection increased the sensitivity of gefitinib by inhibiting the phosphorylation of AKT or ERK in H1975 and PC-9-PIK3CA-M cells. Similar findings were observed *in vivo* in H1975 xenograft mouse model. QKL and TRQ, with *cooling-heat* TCM treatment principle, should be combined with gefitinib in the treatment of NSCLC. Furthermore, *warming-yang* drug SF should be avoided to be used together with EGFR-TKIs.

## 1. Introduction

Epidermal growth factor receptor tyrosine kinase inhibitors (EGFR-TKIs) are the preferred treatment for intermediate to advanced stage non-small cell lung cancer (NSCLC) in patients with *EGFR* gene mutation [1, 2]. However, 20∼30% of these patients obtained de novo resistance to the first-line or second-line EGFR-TKIs [3, 4]. Moreover, even for the exclusively EGFR-mutant advanced NSCLC patients, the median progression-free survival (PFS) was only 9∼13 months for those who received the first-line EGFR-TKI therapy and developed secondary resistance [1, 2]. Therefore, effective therapies to increase the sensitivity to gefitinib for patients in these situations are urgently required.

Traditional Chinese medicine (TCM) therapies have been widely used in cancer treatment including NSCLC in China. Although clinical trials have reported the efficacy of some TCM decoction or patent prescription, with different TCM treatment principles, in combination with EGFR-TKIs, most of them were with small sample size and low quality [5]. What is more, a case report by Hwang et al. indicated that some TCM herbs may even induce resistance to gefitinib [6]. This phenomenon is similar to that in the western medicine that certain drugs, such as antifungal drugs, could induce primary and acquired resistance of EGFR-TKI in lung cancer [7]. Therefore, it is of a great value to explore which TCM treatment principle should be combined with EGFR-TKIs and which principle should be avoided to be combined with EGFR-TKIs using the TCM theory. We have previously demonstrated that EGFR gene mutated NSCLC patients, who are sensitive to EGFR-TKIs, were more likely with *Yin-cold* (YC) TCM syndrome type [8]. Besides, the most common side effects of EGFR-TKIs are red acneiform rashes [1–4, 9], with thirsty, red, and dry tongue and yellow tongue coating, that are typical symptoms and signs of *Yang-heat* (YH) syndrome type. Therefore, EGFR-TKIs may affect the TCM *warming-yang* symptoms. We also noticed that patients with primary or secondary resistance to EGFR-TKIs have more chance to be diagnosed with YH syndrome type. According to TCM theory, we propose a hypothesis that *cooling-heat,* rather than *warming-yang* TCM treatment principle, should be used when combined with EGFR-TKIs and may help to improve the efficacy of EGFR-TKIs in resistant NSCLC.

In order to confirm this hypothesis, we used Qing-kai-ling (QKL) injection/oral solution and Shen-fu (SF) injection/SF decoction, representing typic *cooling-heat* and *warming-yang* drugs, respectively, with no known anticancer effect. QKL is a notable antipyretic preparation and is widely used for the treatment of the upper respiratory inflammation, viral encephalitis, pneumonia, and high fever in clinical practice [10] and is listed in the Chinese Pharmacopoeia (State Pharmacopoeia Committee, 2020). SF has been used for nearly 30 years in China for patients with YC syndrome type. It can attenuate postresuscitation brain edema, myocardial dysfunction, and lung injury [11]. Gefitinib was combined with QKL or SF solution, both *in vitro* and *in vivo*, to detect the interactions and underlying mechanisms. Another classical TCM formulation, Tan-re-qing (TRQ) injection, also termed as *cooling-heat* traditional Chinese medicine, is commonly used to treat acute upper respiratory tract infection and early stage pneumonia in clinical practice [12]. In this study, we used TRQ injection to investigate whether other cooling-heat traditional Chinese medicines had synergic effects with gefitinib. PC-9-PIK3CA-mutation (PC-9-PIK3CA-M, PC-9 with stable overexpression of *PIK3CA* mutated gene, 19Del/PIK3CA) and H1975 (EGFR 19Del/T790M) cell lines were selected for the study, because PIK3CA [13] and T790M [14] were the most important genetic covariations in primary and secondary EGFR-TKIs-resistant patients, respectively.

## 2. Materials and Methods

### 2.1. Preparation of Drugs

Gefitinib was bought from Selleck Co., Ltd (Shanghai, China). QKL injection used *in vitro* was bought from Yi-Sheng Pharmaceutical Co., Ltd (Ji'an, China) and QKL oral solution applied *in vivo* was purchased from Ming-Xing Pharmaceutical Co., Ltd (Guangzhou, China), SF injection used *in vitro* was purchased from Yaan San-Jiu Pharmaceutical Co., Ltd (Sichuan, China), and TRQ injection applied *in vitro* was bought from Kai-Bao Pharmaceutical Co., Ltd (Shanghai, China). Components of QKL include *Isatis tinctoria L.*, *Gardenia jasminoides J.Ellis*, *Lonicera japonica Thunb.*, Concha *Margaritifera Usta, powdered buffalo horn*, baicalin, hyodeoxycholic acid, and cholic acid. The constituents of QKL prescription are shown at Table 1. Different batches of QKL injection were analyzed in consistency studies (chromatograms) using high performance liquid chromatography (HPLC) [15]. SF is prepared by the well-known traditional Chinese herbs *Panax Ginseng* and Radix *Aconiti Carmichaeli*. The consistency of the quality of SF injection over different batches has been ensured by fingerprint technology, thereby identifying the authenticity of drugs [11]. TRQ injection is produced from the raw material of five herbs: *Scutellariae Radix*, *Fel selenarcti*, *Cornu naemorhedi*, *Lonicerae japonicae flos*, and *forsythia fructus*. The consistency of the quality of TRQ injection over different batches has been ensured by a reliable ultra-high performance liquid chromatography-mass spectrometry technology (UHPLC-MS) [16]. The components of herbs of SF decoction applied in the *in vivo* experiments, prepared *Radix Panax Ginseng* and *Radix Aconiti Carmichaeli*, were purchased from Kang-Mei Pharmaceutical Co., Ltd (Guangzhou, China). Seventy-five grams (g) of *Panax Ginseng* and 150 g *Radix Aconiti Carmichaeli* soaked in water were boiled twice; then the mixed solution was concentrated into 250 mL in the rotary evaporator (IKA®RV 10 Basic), with a concentration of 0.9 g/mL crude drug. The constituents of SF prescription are shown at Table 2.

### 2.2. Reagents

4,5-Dimethylthiazol-2, 5-diphenyl-2-H-tetrazolium bromide (MTT) was bought from MP Biomedicals (California, USA). Annexin V/propidium iodide (PI) apoptosis kit was purchased from MultiSciences Biotech Co., Ltd (Hangzhou, China). BCA protein assay kit was bought from Thermo Fisher Scientific, Inc (ML, USA). Rabbit anti-human PIK3CA, EGFR, phospho-EGFR (p-EGFR), AKT, p-AKT, ERK, and p-ERK monoclonal antibodies (mAb) and horseradish peroxidase (HRP) conjugated anti-rabbit antibody were purchased from Cell Signaling Technology, Inc (MA, USA). Electro-Chemi-Luminescence (ECL) reagent was purchased from Millipore Corporation (MA, USA).

### 2.3. Cell Culture

Human PC-9 cells (*EGFR 19Del*) and H1975 cells (*EGFR 19Del/T790M*) were obtained from the Cell Line Bank at the Laboratory Animal Center of Sun Yat-sen University (Guangzhou, China) and the Macao University of Science and Technology (Macao, China), respectively. Cells were maintained in RPMI-1640 medium (Gibco, MA, USA) supplemented with 10% FBS (Gibco, MA, USA) and 1% penicillin-streptomycin sulfate (Gibco, MA, USA) and incubated at 37°C with 5% CO_2_.

### 2.4. The Construction of PC-9-PIK3CA-M Cell Line Using Lentivirus Transfection

The PC-9 cells were planted into a 12-well plate with the cell density of 1 × 10^5^/well. The cells were transfected with 10 MOI of lentivirus containing *PIK3CA* mutation gene (E545K) overexpressing plasmid after 24 h of incubation. When the cells reached 80%–90% confluence, PC-9 cells were passaged and 2 *μ*g/mL puromycin was added to the media for maintenance culture to select the virus infected cells. Then Western blotting analysis was used to confirm the overexpression of PIK3CA and its downstream protein expression in PC-9-PIK3CA-M cells.

### 2.5. Cell Viability Assay

MTT assay was used to measure cell viability. Cells were planted in 96-well culture plates at the density of 5 × 10^3^/well. After 24 h of incubation, cells were treated with drugs alone or in combination for 48 h and 72 h. Then the cells were incubated with 0.5 mg/mL MTT at 37°C for 4 h. The dimethyl sulfoxide (DMSO) was added and the light absorbance at 570 nm of each well was measured spectrophotometrically using a microplate reader (VICTOR X5, PerkinElmer, USA). The cell proliferation inhibition rate was determined as follows: inhibition rate = (OD_570nm_ value of the control group - OD_570nm_ value of the experimental group)/OD_570nm_ value of the control group.

### 2.6. Apoptosis Assay

Cells were planted in 6-well culture plates at a density of 3 × 10^5^ cells/well. After 24 h of incubation, cells were treated with drugs alone or in combination, with the optimal concentration and exposure time according to the MTT assay results. After removing the medium, cells were trypsinized with EDTA-free trypsin solution, harvested, and then resuspended in 500 *μ*L Binding Buffer (1×) with 5 *μ*L Annexin V-FITC and 10 *μ*L PI. After incubation for 5 min at room temperature in the dark, the samples were analyzed using a flow cytometer (Beckman Coulter FC500, USA).

### 2.7. Western Blot Analysis

The cells were plated in 6-well culture plates at a density of 3 × 10^5^ cells/well. After 24 h of incubation, cells were treated with drugs alone or in combination. After 48 h treatment, the cells were lysed in lysis buffer. The concentration of proteins was determined, and 20–30 *μ*g protein of each group was resolved on an 10% denatured SDS-polyacrylamide gel and transferred onto a PVDF membrane (Millipore, MA, USA). After blocking nonspecific binding sites with 5% milk, the membranes were incubated with rabbit anti-human EGFR, p-EGFR, AKT, p-AKT, ERK, and p-ERK monoclonal antibodies overnight at 4°C. Then the membranes were incubated with HRP conjugated anti-rabbit antibody for 1 h at room temperature. Finally, signals were detected using a freshly prepared ECL solution and the ChemiDoc XRS + System (Bio-Rad, Hercules, CA, USA). ImageLab software (version 4.0) was used to calculate the expression of each protein, which was normalized by GAPDH.

### 2.8. Determination of the Antitumor Effect in Nude Mice

The animal experiment was approved by the Animals Research Committee of Guangdong Provincial Hospital of Chinese Medicine (NO.2017050). Sixty Female BALB/c nude mice (18–20 g) were obtained from the Laboratory Animal Center of Southern Medical University (Guangzhou, China, License NO. 44002100006205) and kept in the Animal Center of Guangdong Provincial Hospital of Chinese Medicine (License NO. SYXK (yue) 2013–0094). H1975 cells (6 × 10^6^/mouse) were subcutaneously inoculated into the right forelimb of the nude mice. Tumor growth was measured with the longest diameter (a) and the shortest diameter vertical to a (b). Tumor volume was calculated using the formula, V = *π*ab^2^/6. When the tumors reached the size over 150 mm^3^, the mice were randomly divided into 6 groups (*n* = 10): control (saline solution 0.2 mL), gefitinib (1 mg, in 0.2 mL saline solution), QKL oral solution (0.25 mL), SF decoction (0.2 mL), gefitinib + SF, and gefitinib + QKL group. The compounds were administered by gavage once a day for consecutive 21 days. The daily dosage of each drug for nude mice (with an average weight of 20 g) was obtained based on the daily dosage for human in clinical and the human-mouse transfer formula: Animal dose = Human dose × (HKm/AKm), where HKm and AKm represent the Km factor of human (37) and mouse (3) [17]. The daily dosages for human (with average weight of 60 kg) are 250 mg of gefitinib, 60 ml of QKL oral solution, and 45 g crude drug of SF decoction. The mice were sacrificed 21 days after the treatment using isoflurane and the tumors were excised and weighed and were later placed in paraformaldehyde (4%) for immunohistochemistry (IHC).

### 2.9. IHC

For IHC staining, the sections were applied to block endogenous peroxide activity and then boiled in 0.01 M citrate buffer (pH 6.0) with an autoclave. Tissue sections were incubated with rabbit anti-human EGFR (1 : 100), p-EGFR (1 : 100), AKT (1 : 200), p-AKT (1 : 50), ERK (1 : 200), or p-ERK (1 : 100) monoclonal antibodies at 4°C overnight. The sections were incubated with HRP conjugated anti-rabbit antibody after washing, and the peroxidase reaction was developed with diaminobenzidine substrate kit (Zhongshan Golden Bridge-Bio, Beijing, China). Hematoxylin (Dingguo Changsheng Biotechnology Co., Ltd, Beijing, China) was used for nuclear staining. Image-ProPlus software (version 5) was used to calculate the ratio of integrated optical density (IOD) to area (IOD/Area).

### 2.10. Statistics

Statistical analysis was performed using SPSS 19.0 statistical software (SPSS, Inc., Chicago, USA). The *in vitro* experiments were performed three times, independently. All data were presented as the mean ± standard deviation (SD). Differences between groups were assessed by two-tailed *t*-test, one-way analysis of variance, or analysis of variance for repeated measuring data, and least significant difference (LSD) *t*-test was used for multiple comparisons. *P* < 0.05 was considered to indicate a statistically significant difference. *q value* method was used to evaluate the combination effect of gefitinib and QKL/SF, and it was calculated using the equation: *q* = EAB/(EA + EB- EA×EB), where EA, EB, and EAB were the inhibition effect of gefitinib, QKL/SF, and gefitinib combined with QKL/SF, respectively [18]. A *q value* of 1.15 or more is considered as synergism, *q* < 0.85 as antagonism, and the value between 0.85 and 1.1.5 as additive effect.

## 3. Results

### 3.1. PC-9-PIK3CA-M Cells Activated PIK3CA-AKT Signal Pathway and Became Less Sensitive to Gefitinib due to Upregulated Phosphorylation of AKT Protein

**Figure 1:**
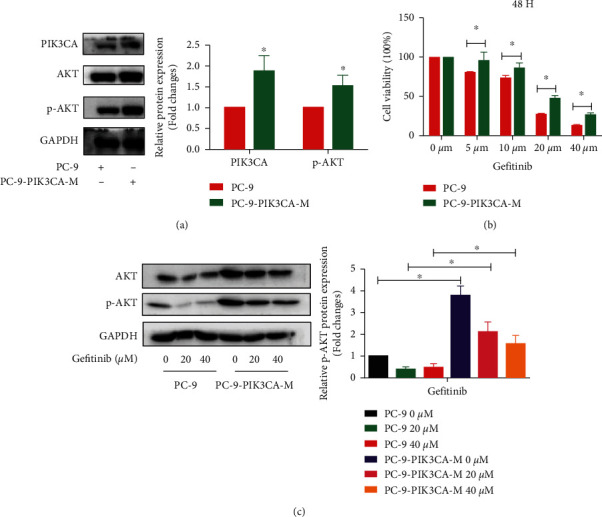
PC-9-PIK3CA-M cells are less sensitive to gefitinib due to the activated PIK3CA-AKT signal pathway: (a) Western blot assay detecting the protein expression of PIK3CA, AKT, and p-AKT in PC-9 and PC-9-PIK3CA-M cells. (b) MTT assay detecting the cell viability after the treatment with gefitinib in PC-9 and PC-9-PIK3CA-M cells. (c) Western blot assay detecting the protein expression of AKT, p-AKT after the treatment with gefitinib in PC-9 and PC-9-PIK3CA-M cells. Data are presented as the mean ± standard deviation of three independent experiments. ^*∗*^*P* < 0.05 vs. control group. PC-9-PIK3CA-M, PC-9-PIK3CA-mutation.

Because there was no NSCLC cell line expressing both *EGFR 19Del* gene and *PIK3CA* mutation gene, we used the lentiviral transfection to stably overexpress *PIK3CA* mutation gene in PC-9 cell line to obtain the PC-9-PIK3CA-M cell model. *PIK3CA* gene mutation activates the PIK3CA/AKT/mTOR signal pathway [19]. The PIK3CA and p-AKT were overexpressed in PC-9-PIK3CA-M cells (Figure 1(a), *P* < 0.05). MTT results indicated that PC-9-PIK3CA-M cells were less sensitive to gefitinib, when compared with PC-9 cells (Figure 1(b); *P* < 0.05). With the same dose treatment of gefitinib, the expression of p-AKT was significantly higher in PC-9-PIK3CA-M cells than that in PC-9 cells (Figure 1(c); *P* < 0.05). These results indicated that PC-9-PIK3CA-M were less sensitive to gefitinib due to upregulated phosphorylation of AKT protein.

### 3.2. QKL Injection and TRQ Injection, but Not SF Injection, Significantly Inhibited the Cancer Cell Viability *In Vitro*

We first screened the concentration of gefitinib. According to the MTT assay, 15 *μ*M of gefitinib for PC-9-PIK3CA-M cells and 25 *μ*M for H1975 cells were used in the experiments with 48 hours of drug exposure, and 15 *μ*M gefitinib for PC-9-PIK3CA-M cells and 20 *μ*M for H1975 cells were used to expose the cells to the drugs for 72 hours.

**Figure 2:**
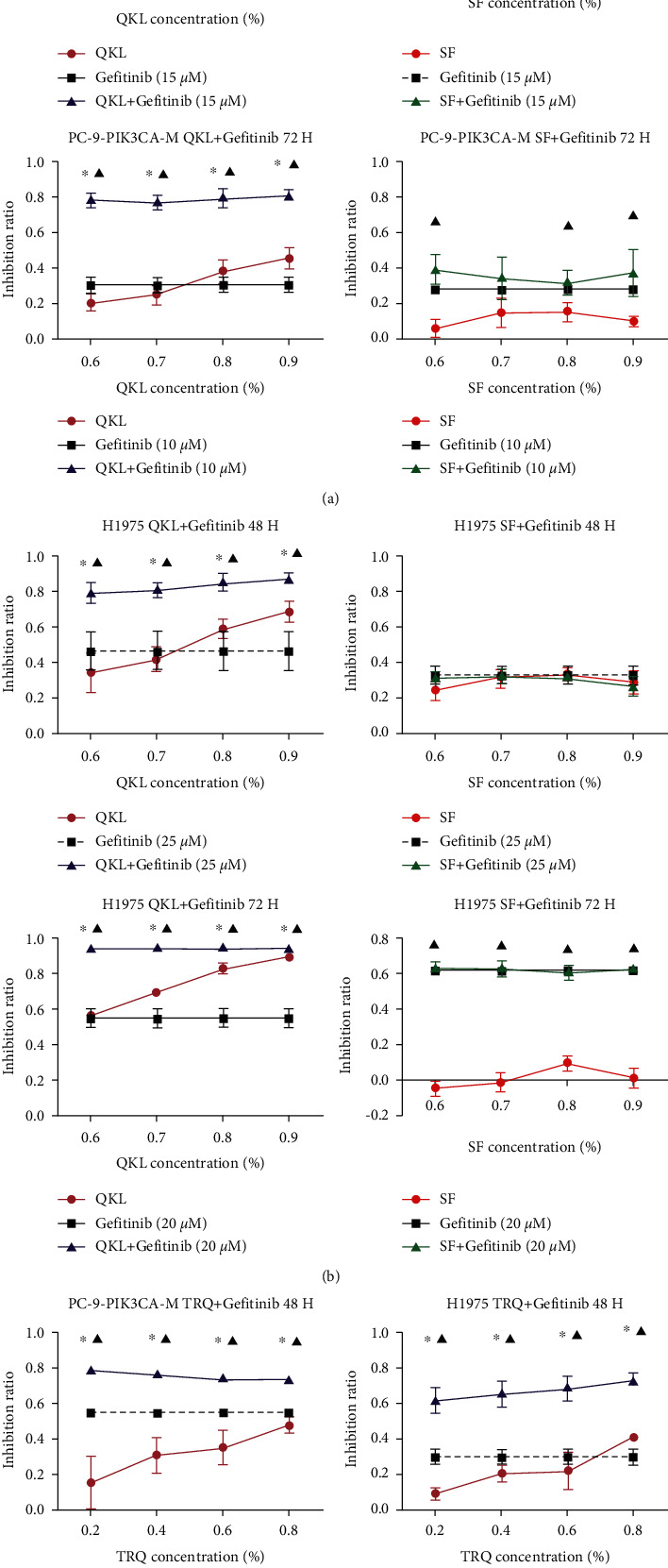
QKL injection and TRQ injection, but not SF injection, increased the sensitivity of gefitinib in PC-9-PIK3CA-M and H1975 cells: (a) MTT assay on the cell viability after the treatment with QKL/SF and gefitinib for 48 h and 72 h in PC-9-PIK3CA-M cells. (b) MTT assay detecting the cell viability after the treatment with QKL/SF and gefitinib for 48 h and 72 h in H1975 cells. (c) MTT assay detecting the cell viability after the treatment with TRQ and gefitinib for 48 h in PC-9-PIK3CA-M and H1975 cells. Data are presented as the mean ± standard deviation of three independent experiments. ^*∗*^*P* < 0.05 vs. gefitinib alone, ^▲^*P* < 0.05 vs. QKL/SF alone. QKL, Qing-kai-ling injection; SF, Shen-fu injection; TRQ, Tan-re-Qing injection.

As shown in Figure 2(a), in PC-9-PIK3CA-M and H1975 cells, the concentration of 0.6%, 0.7%, 0.8%, and 0.9% QKL injection combined with gefitinib induced significantly increased antiproliferation after the treatment for both 48 h and 72 h, when compared with the gefitinib group and QKL group (Figures 2(a) and 2(b); *P* < 0.05). However, there was no significant difference in viability between gefitinib group and SF + gefitinib group in PC-9-PIK3CA-M cells after the treatment with SF at any concentration for both 48 h and 72 h (Figure 2(a); *P* < 0.05). The same phenomenon was observed in H1975 cells (Figure 2(b); *P* < 0.05). Moreover, it was confirmed that another *cooling-heat* traditional Chinese medicine, TRQ injection, also had synergy effect with gefitinib (Figure 2(c); *P* < 0.05).

### 3.3. QKL Injection Synergistically Increased the Proapoptotic Effect of Gefitinib, While SF Injection Antagonized the Proapoptotic Effect of Gefitinib *In Vitro*

**Figure 3:**
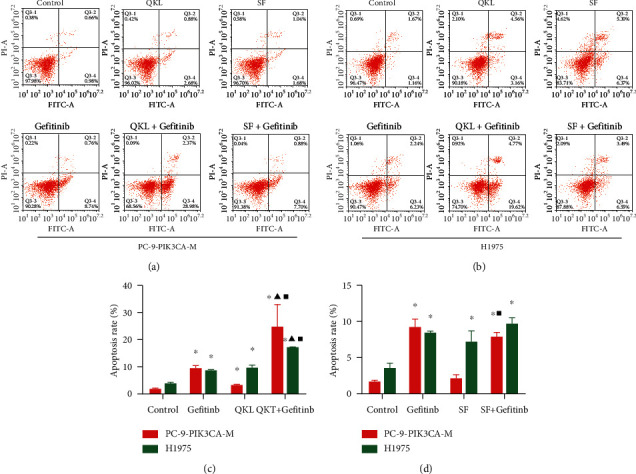
QKL injection increased, and SF injection antagonized the proapoptotic effect of gefitinib in PC-9-PIK3CA-M and H1975 cells: (a) apoptosis assay to assess the combination effect of gefitinib and QKL/SF for 48 h in PC-9-PIK3CA-M cells. (b) Apoptosis assay to assess the combination effect of gefitinib and QKL/SF for 48 h in H1975 cells. (c) The statistics of apoptotic rate induced by gefitinib and QKL in PC-9-PIK3CA-M and H1975 cells. (d) The statistics of apoptotic rate induced by gefitinib and SF in PC-9-PIK3CA-M and H1975 cells. Data are presented as the mean ± standard deviation of three independent experiments. ^*∗*^*P* < 0.05 vs. control group, ^▲^*P* < 0.05 vs. gefitinib group, and ^■^*P* < 0.05 vs. QKL/SF group. QKL, Qing-kai-ling injection; SF, Shen-fu injection.

QKL + gefitinib group significantly increased the rate of apoptosis, when compared with the control, gefitinib, and QKL groups in both the PC-9-PIK3CA-M and H1975 cells (Figure 3; *P* < 0.05). However, the differences in apoptotic rates were not significant between the SF + gefitinib group and gefitinib group (Figure 3; *P* < 0.05) in PC-9-PIK3CA-M cells as well as in H1975 cells. Synergism effect of QKL injection and gefitinib on apoptosis was seen in PIK3CA-M cells (*q* = 2.08), and QKL injection and gefitinib have additive effect in H1975 cells (*q* = 0.99) using the *q value* method. On the contrary, SF injection antagonized the proapoptotic effect of gefitinib in both PIK3CA-M (*q* = 0.71) and H1975 cells (*q* = 0.64).

### 3.4. QKL/TRQ Injection, but Not SF Injection, in Combination with Gefitinib Inhibits the AKT or ERK Protein Expression *In Vitro*

**Figure 4:**
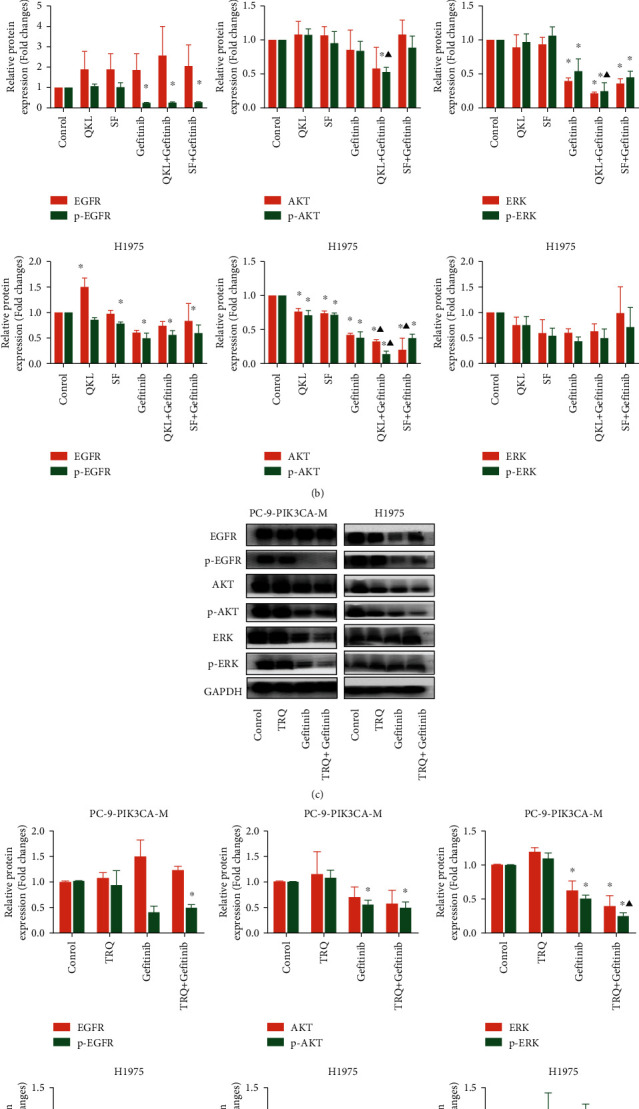
QKL/TRQ injection, not SF injection, enhanced the inhibition of the downstream signaling pathways of EGFR induced by gefitinib *in vitro*. (a) Western blot showing the expression of proteins related to the combination effect of gefitinib and QKL/SF in PC-9-PIK3CA-M and H1975 cell lines. (b) The effects of gefitinib and QKL/SF on EGFR, p-EGFR, AKT, p-AKT, ERK, and p-ERK protein expression in PC-9-PIK3CA-M cells and H1975 cells. (c) Western blot showing the expression of proteins related to the combination effect of gefitinib and TRQ in PC-9-PIK3CA-M and H1975 cell lines. (e) The effects of gefitinib and TRQ on EGFR, p-EGFR, AKT, p-AKT, ERK, and p-ERK protein expression in PC-9-PIK3CA and H1975 cells. Data are presented as the mean ± standard deviation of three independent experiments. ^*∗*^*P* < 0.05 vs. control. ^▲^*P* < 0.05 vs. gefitinib alone. QKL, Qing-kai-ling injection; SF, Shen-fu injection; TRQ, Tan-re-qing injection.

The protein expression levels of p-AKT were significantly lower in gefitinib + QKL group in PC-9-PIK3CA-M cells, comparing with the control group and the gefitinib group (Figure 4; *P* < 0.05). No significant differences were seen in p-AKT expression among the control group, gefitinib, and gefitinib + SF groups. Besides, the protein expression of p-ERK was significantly lower in gefitinib + QKL group in PC-9-PIK3CA-M cells, comparing with the control group and the gefitinib group (Figure 4; *P* < 0.05). Besides, no significant differences were seen in the p-ERK expression between the gefitinib and gefitinib + SF groups. These results suggested that the downregulated phosphorylation of AKT and ERK protein may contribute to the synergic effect of QKL injection and gefitinib in PC-9-PIK3CA-M cells. In H1975 cell line, the p-AKT protein expression was much lower in QKL + gefitinib group, when compared with both the control group and the gefitinib group (Figure 4; *P* < 0.05). There was no significant difference seen in the p-AKT expression between SF + gefitinib group and gefitinib group. However, the expression of EGFR, p-EGFR, AKT, ERK, and p-ERK has no significant difference in the gefitinib, QKL + gefitinib, and SF + gefitinib groups. These results suggested that the downregulation of p-AKT may contribute to the synergistic effect between QKL injection and gefitinib in H1975 cells. These results suggested that the downregulated phosphorylation of AKT protein may contribute to the synergic effect of QKL injection and gefitinib *in vitro*.

Furthermore, to examine whether other cooling-heat traditional Chinese medicines, other than QKL, have inhibitory effects on EGFR/AKT and EGFR/ERK signaling pathways, we detected the effects of TRQ injection and gefitinib on the expression of EGFR, AKT, and ERK proteins. The results indicated that the downregulated phosphorylation of ERK protein contributed to the synergism effect between QKL injection and gefitinib in PC-9-PIK3CA-cells, while the downregulated phosphorylation of AKT protein might contribute to the synergic effect of QKL injection and gefitinib in H1975 cells.

### 3.5. QKL Oral Solution, Not SF Decoction, Enhanced the Antitumor Effect of Gefitinib via Regulating p-AKT *In Vivo*

**Figure 5:**
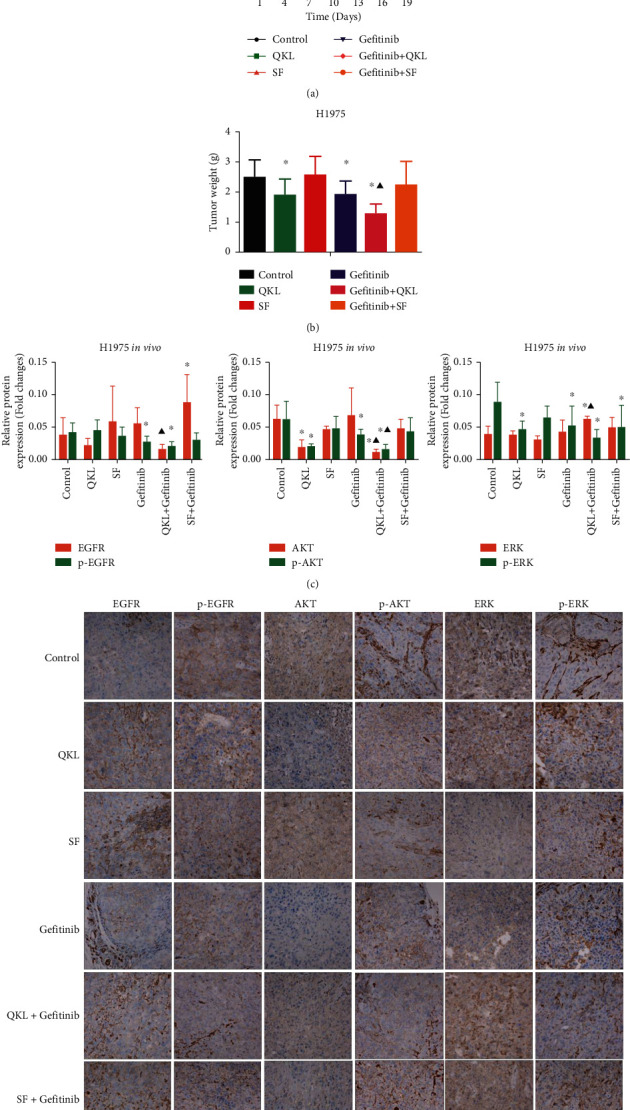
QKL oral solution, not SF decoction, and gefitinib synergistically inhibited the tumor growth via regulating p-AKT *in vivo*. (a) The tumor growth curve showing the anticancer effect of gefitinib and QKL/SF in H1975 xenograft transplanted nude mice. (b) The tumor weight showing the anticancer effect of gefitinib and QKL/SF in H1975 xenograft transplanted nude mice at day 19. (c) The statistics of EGFR, p-EGFR, AKT, p-AKT, ERK, and p-ERK protein expression in H1975 xenograft transplanted nude mice. (d) Immunohistochemical staining showing the effects of gefitinib and QKL/SF on the expression of EGFR, p-EGFR, AKT, p-AKT, ERK, and p-ERK protein (×400 magnifications). ^*∗*^*P* < 0.05 vs. control group. ^▲^*P* < 0.05 vs. gefitinib group. QKL, Qing-kai-ling oral solution; SF, Shen-fu decoction.

QKL, gefitinib, and QKL + gefitinib inhibited tumor growth with significantly smaller tumor volume and lighter tumor weight, compared with those in control group (Figures 5(a) and 5(b); *P* < 0.05). There was a significant difference in tumor volume and tumor weight between QKL + gefitinib group and gefitinib group (Figures 5(a) and 5(b); *P* < 0.05). However, there was no significant difference between SF + gefitinib group and gefitinib group, or SF + gefitinib group and control group.

In H1975 xenograft transplanted nude mice, the p-AKT protein expression was much lower in the QKL + gefitinib group compared with that in the control group (Figures 5(c) and 5(d); *P* < 0.05) as well as the gefitinib group (Figures 5(c) and 5(d); *P* < 0.05). There was no significant difference in the p-AKT protein expression between SF + gefitinib group and gefitinib group, or SF + gefitinib group and control group. These results demonstrated that the downregulated phosphorylation of AKT may contribute to the additive effect of QKL and gefitinib *in vivo* [18].

## 4. Discussion

TCM has been widely used in at least 78 countries [20], especially in East Asia. Many NSCLC patients receive TCM treatments when taking EGFR-TKIs, with the wish to delay or reverse EGFR-TKI resistance. A meta-analysis on a biomedical literature database showed that using TCM in combination with EGFR-TKIs was significantly superior to the use of EGFR-TKI alone in total response rate, quality of life improvement, and one-year survival rate of patients with NSCLC (*P* < 0.05) [21]. TCM combination therapy may increase therapeutic effects and reduce toxicity when combined with EGFR-TKIs for advanced NSCLC as well, indicating that this combination maximizes the duration of the EGFR- TKI treatment for NSCLC patients. However, inappropriate use of TCM may antagonize the effect of EGFR-TKIs [6]. In a case report, the NSCLC patient was treated with gefitinib as the first-line treatment and had progressively increasing shortness of breath and imaging examination revealed progression of her disease within 9 weeks of gefitinib treatment [6]. Later on, it was found that multiple complementary herbal medicines such as ginseng, taken with gefitinib by the patients without the permission of the doctor, led to the disease progression, and her disease was finally relieved after taking gefitinib alone for 30 weeks [6]. In the real world of TCM clinical practice, clinicians first differentiate TCM syndrome types, then decide the TCM treatment principle, and finally write out a TCM prescription. Therefore, deciding TCM treatment principle is an important step, acting as bridge between TCM syndrome diagnosis and final TCM prescription. For this reason, we have focused on finding out the appropriate TCM treatment principles in the combinational use of EGFR-TKIs, as well as the inappropriate TCM treatment principles to avoid when EGFR-TKIs are used in the treatment of NSCLC.

According to our finding, it seems that *cooling-heat*, rather than *warming-yang* TCM treatment principle, should be used when combined with EGFR-TKIs. Firstly, it was found that NSCLC patients with *YC* TCM syndrome were more likely to have the *EGFR* gene mutations than those with *YH* syndrome based on our previously published data [8]. Other team also found that *heat* syndrome was one of the most common syndromes in the NSCLC patients with *EGFR* gene mutation [22]. Since EGFR-TKIs are used in EGFR-mutated patients who are more common with YC syndrome, it might affect some *warming-yang* TCM syndrome. The most common side-effect of EGFR-TKIs, red acneiform rashes, also indicated its *warming-yang* effect. Because of the long-term need for *warming-yang* EGFR-TKIs taking, herbs with clear-heat function should be combined to get the balance between *Yin* and *Yang* and warm-yang herbs need to be avoided in case of the aggravation of YH syndrome type. Second, we also found that toxic-heat TCM syndrome was significantly associated with resistance to EGFR-TKI treatment (median PFS, 5.13 *vs.* 10.2 months). According to TCM theory, *clearing-heat* TCM herbs should be used in these toxic-heat patients, while *warm-yang* herbs should be avoided in this situation. Moreover, many TCM oncologists also use *clearing-heat* TCM decoctions to increase the effectiveness and reduce toxicity of EGFR-TKIs in clinical practice [23]. It was found that the classic *clearing-heat* TCM decoctions Yin-Qiao powder could significantly improve the skin rashes caused by EGFR-TKIs [24]. Compared with EGFR-TKIs alone, the *clearing-heat* TCM decoctions and EGFR-TKIs could significantly improve the patients' clinical curative effect [25]. The present study demonstrated that, in gefitinib resistant *T790 M* or *PIK3CA* comutated models, *cooling-heat* TCM prescription QKL and TRQ, but not *warming-yang* prescription SF solution, could significantly enhance the antitumor effects of gefitinib both *in vitro* and *in vivo.* Surprisingly, SF injection even antagonized the proapoptotic effect of gefitinib *in vitro.*

We also tried to find out the underlying mechanism of the synergic effect of QKL and TRQ to gefitinib. EGFR signaling is critical for several cellular functions including survival, proliferation, differentiation, and motility of cancer cells. EGFR activation transduces multiple signaling pathways, including the RAS/RAF/ERK pathway and the PI3K/AKT/mTOR pathway [26]. Our findings demonstrated that AKT phosphorylation and ERK phosphorylation inhibition served an important role in the synergic effect of QKL/TRQ and gefitinib in PC-9-PIK3CA-M and H1975 cells *in vitro* and H1975 xenograft nude mice *in vivo*. Researchers also found that *clearing-heat* TCM decoctions or active ingredients from *clearing-heat* herbs can inhibit the growth of lung cancer via RAS/RAF/ERK pathway and the PI3K/AKT/mTOR pathway. The *cooling-heat* medicine Compound Kushen injection significantly improved the sensitivity of gefitinib on less sensitive NSCLC cells in a combinatorial fashion through the PI3K/AKT/mTOR signaling pathway [27]. Solamargine, derived from *cooling-heat* medicine *Solanum nigrum L*, inhibited the growth of human lung cancer cells through inactivation of AKT signaling [28]. Ophiopogonin B, a saponin extracted from *Ophiopogon japonicus* (Thunb.) Ker Gawl., the *cooling-heat* herb, could suppress epithelial-mesenchymal transition through AKT phosphorylation [29]. Regulating the downstream signaling pathways of EGFR may act as important mechanisms underlying the *cooling-heat* medicine in exerting anticancer function in lung cancer.

There were several limitations in our present study. Firstly, we only used one or two drugs to present each of the treatment principles. Expansion of the observed synergic effect of QKL/TRQ, but not SF, with gefitinib to the effect of *cooling-heat* or *warming-yang* treatment principle should be cautious. We should further use other TCM patent prescriptions or decoction to present the *cooling-heat* or *warming-yang* treatment principle. Secondly, NSCLC cell lines with different EGFR-TKIs-resistant types should be applied to detect the combination effect of gefitinib and *cooling-heat*/*warming-yang* drugs. All these researches will be meaningful for guiding the principle of TCM therapies in combination with EGFR-TKIs or avoiding using them together with EGFR-TKIs to delay the resistance to EGFR-TKIs or increase the sensitivity of EGFR-TKIs in EGFR-TKIs-resistant NSCLC models.

## 5. Conclusions

In conclusion, QKL or TRQ, with *cooling-heat* TCM treatment principle, not *warming-yang* drug SF, increases the efficacy of gefitinib in the resistant NSCLC models. Furthermore, SF should be avoided to be used together with EGFR-TKIs. The influence of other drugs with *cooling-heat* or *warming-yang* TCM treatment principle warrants further study.

## Figures and Tables

**Table 1:** Constituents of Qing-kai-ling prescription.

Common name	Weight (g)
Isatidis radix	200.00
Lonicerae japonicae flos	60.00
Gardeniae fructus	25.00
Buffalo horn	25.00
Concha margaritifera	50.00
Baicalin	5.00
Cholic acid	3.25
Hyodeoxycholic acid	3.75

**Table 2:** Constituents of Shen-Fu prescription.

Common name	Weight (g)
Radix ginseng	75.00
Radix aconiti lateralis preparata	150.00

## Data Availability

The data used in the study are available upon request by writing to the corresponding author.

## Abbreviations

EGFR-TKIs: Epidermal growth factor receptor tyrosine kinase inhibitors
NSCLC: Non-small cell lung cancer
PC-9-PIK3CA-M: PC-9-PIK3CA-mutation
QKL: Qing-kai-ling
SF: Shen-fu
TRQ: Tan-re-qing
HPLC: High performance liquid chromatography
UHPLC-MS: Ultra-high performance liquid chromatography-mass spectrometry technology
PFS: Progression-free survival
TCM: Traditional Chinese medicine
YC: *Yin-cold*
YH: *Yang-heat*
ORR: Objective response rate.
