# Erratum to “Qing-Kai-Ling Injection Acts Better Than Shen-Fu Injection in Enhancing the Antitumor Effect of Gefitinib in Resistant Non-Small Cell Lung Cancer Models”

**DOI:** 10.1155/2022/9808974

**Published:** 2022-03-18

**Authors:** Ya-Ya Yu, Yan-Juan Zhu, Ying Zou, Zhen-Zhen Xiao, Shuai Shi, Yi-Hong Liu, Xue-Song Chang, Ya-Dong Chen, Hai-Bo Zhang

**Affiliations:** ^1^Department of Oncology, The Second Affiliated Hospital of Guangzhou University of Chinese Medicine, Guangzhou, China; ^2^Department of Oncology, Guangdong Provincial Hospital of Chinese Medicine, Guangzhou, China; ^3^Guangdong-Hong Kong-Macau Joint Lab on Chinese Medicine and Immune Disease Research, Guangzhou, China; ^4^Guangdong Provincial Key Laboratory of Clinical Research on Traditional Chinese Medicine Syndrome, Guangzhou, China; ^5^Department of Integrated Traditional and Western Medicine, Affiliated Cancer Hospital and Institute of Guangzhou Medical University, Guangzhou, Guangdong, China; ^6^Department of Respiration Medicine, Luoyang First People's Hospital, Luoyang, Henan, China; ^7^State Key Laboratory of Dampness Syndrome of Chinese Medicine, Guangzhou, China

In the article titled “Qing-Kai-Ling Injection Acts Better Than Shen-Fu Injection in Enhancing the Antitumor Effect of Gefitinib in Resistant Non-Small Cell Lung Cancer Models” [1], the author's contributions statement was missing. The correct Authors' Contributions section is as follows:

Ya-Ya Yu and Yan-Juan Zhu contributed equally to this work. Ya-ya Yu and Zhen-zhen Xiao performed the biological experiments in vitro. Ying Zou and Shuai Shi performed the biological experiments in vivo. Yi-hong Liu, Xue-song Chang, and Ya-dong Chen did the statistical analysis. Hai-bo Zhang conceived and supervised the study. Yan-juan Zhu and Ya-ya Yu drafted the manuscript. All the authors read and approved the final manuscript.

The error was introduced during the production process of the article, and Hindawi apologises for causing this error in the article.
